# Deep Sequencing-Identified Kanamycin-Resistant *Paenibacillus* sp. Strain KS1 Isolated from Epiphyte *Tillandsia usneoides* (Spanish Moss) in Central Florida, USA

**DOI:** 10.1128/genomeA.01523-16

**Published:** 2017-02-02

**Authors:** Pushpa Lata, Subramaniam S. Govindarajan, Feng Qi, Jian-Liang Li, Malaya K. Sahoo

**Affiliations:** a Department of Microbiology and Molecular Biology, University of Central Florida, Orlando, Florida, USA; b Analytical Genomics Core, Sanford Burnham Prebys Medical Discovery Institute at Lake Nona, Orlando, Florida, USA; c Applied Bioinformatics Core, Sanford Burnham Prebys Medical Discovery Institute at Lake Nona, Orlando, Florida, USA; d Department of Pathology, Stanford University School of Medicine, Palo Alto, California, USA

## Abstract

*Paenibacillus* sp. strain KS1 was isolated from an epiphyte, *Tillandsia usneoides* (Spanish moss), in central Florida, USA. Here, we report a draft genome sequence of this strain, which consists of a total of 398 contigs spanning 6,508,195 bp, with a G+C content of 46.5% and comprising 5,401 predicted coding sequences.

## GENOME ANNOUNCEMENT

*Tillandsia usneoides* (Spanish moss) is an epiphyte that grows luxuriantly in the central Florida region of the United States. *T. usneoides* is a medicinal plant and is also used as an air pollution indicator (1–3). We isolated a bacterium *Paenibacillus* sp. strain KS1 from this epiphyte *T. usneoides* which exhibits high level resistance toward an antibiotic, kanamycin (>1 mg/mL). *Paenibacillus* is a Gram-positive bacterium belonging to phylum *Firmicutes*. Here, we report the whole-genome sequence of *Paenibacillus* sp. strain KS1. The genomic DNA of *Paenibacillus* sp. strain KS1 was isolated using the QIAamp DNA minikit (Qiagen, USA), according to the manufacturer’s instructions. DNA quality was checked by a NanoDrop spectrophotometer (Thermo Scientific) and quantitated by a Qubit 2.0 fluorometer (Life Technologies). The genomic DNA was fragmented and tagged with sequencing adapters in a single tube enzymatic reaction using the Nextera XT DNA library preparation kit (Illumina Inc., CA). The resulting library was sequenced using the Illumina MiSeq sequencing platform (Illumina Inc., CA) and generated 3.6 million paired-end reads. The reads were assembled using SPAdes version 3.1.0 (4). The reads were assembled in the “only-assembler” mode with default k-mer and other parameters. A total of 398 contigs were obtained and the *N*_50_ of the contigs was 61,402 bp. The maximum length of the contig was 185,639 bp with total length of 6,508,195 bp genome. The G+C content of the genome was 46.5%. The protein-coding regions were predicted by the NCBI Prokaryotic Genome Annotation Pipeline (release 2013; https://www.ncbi.nlm.nih.gov/genome/annotation_prok/). In total, 5,401 coding genes were identified for a total length of 6,508,195 bp and 86.94% coverage of the *Paenibacillus* genome. Fourteen tRNA genes, seven rRNA (three 16S rRNA; four 23S rRNA), and four noncoding RNA (ncRNA) genes have putative functions assigned on the basis of the annotation.

### Accession number(s).

This whole-genome shotgun project has been deposited at DDBJ/ENA/GenBank under the accession no. MAIS00000000. The version described in this paper is MAIS01000000.
